# Elevated levels of autoantibodies against EXD2 and PHAX in the sera of patients with chronic thromboembolic pulmonary hypertension

**DOI:** 10.1371/journal.pone.0211377

**Published:** 2019-02-13

**Authors:** Akira Naito, Takaki Hiwasa, Nobuhiro Tanabe, Takayuki Jujo Sanada, Toshihiko Sugiura, Ayako Shigeta, Jiro Terada, Hirotaka Takizawa, Koichi Kashiwado, Seiichiro Sakao, Koichiro Tatsumi

**Affiliations:** 1 Department of Respirology, Graduate School of Medicine, Chiba University, Chiba, Japan; 2 Department of Advancing Research on Treatment Strategies for Respiratory Disease, Graduate School of Medicine, Chiba University, Chiba, Japan; 3 Department of Biochemistry and Genetics, Graduate School of Medicine, Chiba University, Chiba, Japan; 4 Department of Neurological Surgery, Graduate School of Medicine, Chiba University, Chiba, Japan; 5 Department of Advanced Medicine in Pulmonary Hypertension, Graduate School of Medicine, Chiba University, Chiba, Japan; 6 Port Square Kashiwado Clinic, Kashiwado Memorial Foundation, Chiba, Japan; 7 Department of Neurology, Kashiwado Hospital, Kashiwado Memorial Foundation, Chiba, Japan; Kurume University School of Medicine, JAPAN

## Abstract

While circulating autoantibodies have been detected in patients with several cardiovascular diseases, such studies have not been performed for chronic thromboembolic pulmonary hypertension (CTEPH) and pulmonary arterial hypertension (PAH). Here we investigated the production of certain auto-antibodies in CTEPH patients. Initial screening was performed in 5 CTEPH patients and 5 healthy donors (HDs) using a ProtoArray Human Protein Microarray v5.1 containing 9,375 human proteins, and we selected 34 antigens recognized by IgG antibodies more strongly in the sera of CTEPH patients than in the sera of HDs. In subsequent second/third analyses, we validated the auto-antibody level using amplified luminescent proximity homogeneous assay-linked immunosorbent assay (AlphaLISA) in 96 CTEPH patients and 96 HDs as follows: At the second screening, we used 63 crude peptides derived from those selected 34 antigens and found that the serum levels of autoantibodies for 4 peptides seemed higher in CTEPH patients than in HDs. In third analysis, we used the purified peptides of those selected in second screening and found that serum antibodies against peptides derived from exonuclease 3'-5' domain-containing 2 (EXD2) and phosphorylated adaptor for RNA export (PHAX) were significantly higher in CTEPH patients than in HDs. The serum antibody levels to these antigens were also elevated in PAH patients. The titers against EXD2 peptide decreased after surgical treatment in CTEPH patients. These autoantibodies may be useful as biomarkers of CTEPH and PAH, and further investigations may provide novel insight into the etiology.

## Introduction

Chronic thromboembolic pulmonary hypertension (CTEPH) is a form of pulmonary hypertension (PH) caused by persistent thromboemboli of the pulmonary arteries. Various etiological factors, including infection, inflammation, genetic susceptibilities, and insufficient angiogenesis [1], have been discussed as important pathogenetic factors [2]. However, the etiology of CTEPH is not completely understood, and disease-specific, non-invasive biomarkers have not been identified.

Circulating autoantibodies have been detected in patients with several cardiovascular diseases, such as atherosclerosis [3, 4] and other cardiovascular diseases, including coronary artery diseases[5]. As a typical example, anti-phospholipid antibodies reportedly enhance the uptake of oxidized LDL by macrophages, which leads to foam cell formation [5–7]. Recently, we established the auto-antibody screening method using an amplified luminescent proximity homogeneous assay-linked immunosorbent assay (AlphaLISA) and found that anti-adiponectin antibody levels were significantly higher in patients with coronary artery disease, cerebral infarction and diabetes mellitus than in HDs [8]. However, autoantibodies in the context of CTEPH and pulmonary arterial hypertension (PAH) have not yet been thoroughly explored.

In the present study, we comprehensively screened autoantigens recognized by IgG antibodies in the sera of patients with CTEPH using a protein array. We then selected and identified the autoantibodies elevated in the sera of CTEPH patients and also investigated whether or not PAH patients had the same autoantibodies.

## Materials and methods

### Ethical statement

The protocol for the analysis of the sera from CTEPH and PAH patients was approved by the Local Ethical Review Board of the Chiba University Graduate School of Medicine (approval number 1248). The protocol for the serum analysis in healthy donors (HDs) and the patients with sleep apnea syndrome (SAS) was also approved by the Local Ethical Review Board of the Chiba University Graduate School of Medicine (approval number 973). Written informed consent was obtained from all participating patients before sera were collected.

### Patients and healthy donor sera

We collected serum samples from patients diagnosed with CTEPH and PAH at Chiba University Hospital between 2001 and 2015. Serum samples were collected from HDs who underwent annual medical checkups at Port Square Kashiwado Clinic. We also collected serum samples from patients with SAS, as previously reported [9] [10]. Each serum sample was centrifuged at 3,000 × *g* for 10 min at room temperature, and then the supernatant was stored at -80°C until use (no other freeze-thaw cycles).

### The ProtoArray human protein microarrays analysis

Serum samples from 5 CTEPH patients and 5 HDs were profiled on a ProtoArray Human Protein Microarrays v5.1 containing 9,375 human proteins. The serum samples were profiled at a 1:500 dilution, utilizing one ProtoArray Human Protein Microarray per sample. Alexa Fluor 647-anti-human IgG detection reagent was used to quantify the IgG level of associated auto-antibodies. Pairwise comparisons were made between the two sample populations. Assays were performed by Thermo Fisher Scientific (Waltham, MA, USA) according to the manufacturer’s instructions.

### Epitope prediction and peptide synthesis

Possible epitope sites in the selected antigenic proteins were predicted using the software program ProPred (http://www.imtech.res.in/raghava/propred/) as described previously [11].

### Amplified luminescence proximity homogeneous assay (AlphaLISA)

AlphaLISA was performed in 384-well microtiter plates (white opaque ProxiPlate PerkinElmer, Waltham, MA, USA) containing 2.5 μL of 1/100-diluted sera and 2.5 μL of GST or a GST-fusion protein (10 μg/mL) in AlphaLISA buffer (25 mM 4-[2-hydroxyethyl]-1-piperazineethanesulfonic acid [HEPES], pH 7.4, 0.1% casein, 0.5% Triton X-100, 1 mg/mL dextran-500, and 0.05% Proclin-300). The resulting reaction mixture was incubated at room temperature for 6 to 8 h. After incubation, anti-human IgG-conjugated acceptor beads (2.5 μL of 40 μg/mL) and streptavidin-conjugated donor beads (2.5 μL of 40 μg/mL) were added, and the samples were subjected to an additional 7 to 21 days of incubation at room temperature in the dark. The chemical emissions of samples were read on an EnSpire Alpha microplate reader (PerkinElmer). Specific reactions were calculated by subtracting the alpha values of buffer control samples from those of samples containing biotinylated peptides. The details have been described previously [9, 11–14].

### Statistical analyses

All statistical analyses were performed with a commercially available software program (JMP 10.0.2, Japanese version; SAS Institute Tokyo, Japan). The results are expressed as the mean ± standard deviation for continuous variables and as the number and percentage for categorical variables unless otherwise indicated. Comparisons between two groups were performed by Mann-Whitney U test, correlation analysis between two continuous variables were performed by Spearman’s correlation analysis, and multiple comparisons were performed by Steel-Dwass test.

## Results

### Initial screening of CTEPH-specific antigens by a protein array

Using a ProtoArray loaded with 9375 proteins, we examined the sera from 5 CTEPH patients and 5 HDs to identify CTEPH-associated antigens. We selected the antigens for which the absorbance ratio (CTEPH:HD) exceeded 1.5. As a result, the 34 proteins that were more strongly recognized by IgG antibodies in the sera of CTEPH patients than in HDs were selected (Table 1).

**Table 1:** Protein array-selected antigens recognized by serum antibodies of CTEPH patients.

Name	
GATA3	C12orf45
NOL12	APOBEC4
MGRN1	LASP1
FSTL4	AIF1
C11orf54	STAU1
C9orf163	EXD2
C14orf93	TSC22D3
TNFSF13	Bystin
NCF4	XTP3TPA
LCORL	NR0B1
HNRPA0	MYL2
MXI1	PHAX
PDGFRA	SCD
COMMD10	FGF16
CASK	FGF1
PSMA4	IFNG
TCN2	MAPKAPK3

List of protein antigens selected from the first screening using ProtoArray with sera from CTEPH patients and HDs.

### Second screening using crude peptides

The amino acid sequence of the 63 peptides shown in Table 2 were predicted as epitope sites of the 34 candidate antigen proteins selected in the first screening using ProtoArray.

**Table 2:** Amino acid sequences of synthetic peptides used for the second screening.

No.	Name	Sequence	No.	Name	Sequence
1	bGATA3-12	RNSVEIKKKLGLIFKWTN	33	bC12orf45-106	PHSKVIQMDVALFEMNQS
2	bNOL12-88	LVTAKTESVQYDHPNHTV	34	bAPOBEC4-175	RSLASLWPRVVLSPISGG
3	bMGRN1-60	LNFLGSRPVQFPYVTPAP	35	bAPOBEC4-312	KPRNIVRHLNMPQMSFQE
4	bMGRN1-194	RGVFPVVIQAVVDEGDVV	36	bAPOBEC4-331	KDLGRLPTGRSVEIVEIT
5	bMGRN1-245	RVSYLLQEIYGIENKNNQ	37	bLASP1-61	SFTMVADTPENLRLKQQS
6	bFSTL4-147	RLKNVLLALQTRLQPLQE	38	bAIF1-55	MMLGKRSAILKMILMYEE
7	bFSTL4-234	TLREFYIAFQVVQLSLAP	39	bSTAU1-131	HMKNFVTKVSVGEFVGEG
8	bFSTL4-509	VRNRYIYVAQPALSRVLV	40	bSTAU1-165	LEELKKLPPLPAVERVKP
9	bFSTL4-526	VVDIQAQKVLQSIGVDPL	41	bSTAU1-442	CSSQPPLISHGIGKDVES
10	bC11orf54-70	PLVNQKKVYDLNKIAKEI	42	bEXD2-60	GCLDLRYLAMRQRNNLLC
11	bC9orf163-64	RRLVREGVISVPRQQGRR	43	bEXD2-307	KNVIPHEYRKHFPIEMKD
12	bC14orf93-198	YRLFANRSSIMRHFGPED	44	bEXD2-446	GLRSLMQLESRWRQHFLD
13	bTNFSF13-49	LTQQTELQSLRREVSRLQ	45	bTSC22D3-3	LVKNHLMYAVREEVEILK
14	bTNFSF13-156	RIQDAGVYLLYSQVLFQD	46	bBystin-165	TCTLREAIIVGSIITKCS
15	bTNFSF13-193	RCIRSMPSHPDRAYNSCY	47	bBystin-264	KEALLELLRLQPHPQLSP
16	bNCF4-192	KAGDVIFLLSRINKDWLE	48	bXTP3TPA-22	RFSFSPEPTLEDIRRLHA
17	bNCF4-298	LSDEDVALMVRQARGLPS	49	bNR0B1-273	PCFQVLPLDQQLVLVRNC
18	bLCORL-159	ELMKKMIRQFAIEYISKS	50	bNR0B1-430	NSTLFLLRFINANVIAEL
19	bHNRPA0-34	TDCVVVVNPQTKRSRCFG	51	bMYL2-114	LKADYVREMLTTQAERFS
20	bHNRPA0-150	DAADKAAVVKFHPIQGHR	52	bPHAX-247	ARVVRIIGNKKAIELLME
21	bMXI1-1	MERVKMINVQRLLEAAEF	53	bSCD-134	LRLFLIIANTMAFQNDVY
22	bMXI1-85	LCLERLKVLIPLGPDCTR	54	bSCD-250	TFLRYAVVLNATWLVNSA
23	bPDGFRA-10	VLGCLLTGLSLILCQLSL	55	bFGF16-92	GILEFISLAVGLISIRGV
24	bPDGFRA-30	ILPNENEKVVQLNSSFSL	56	bFGF16-152	YKHSDSERQYYVALNKDG
25	bCOMMD10-135	ETVGWQLNLQMAHSAQAK	57	bFGF1-58	QLQLSAESVGEVYIKSTE
26	bCASK-34	ETGQQFAVKIVDVAKFTS	58	bFGF1-138	THYGQKAILFLPLPVSSD
27	bCASK-103	EIVKRADAGFVYSEAVAS	59	bIFNG-32	ILKNWKEESDRKIMQSQI
28	bCASK-254	VRRMLMLDPAERITVYEA	60	bIFNG-101	TDLNVQRKAIHELIQVMA
29	bPSMA4-82	DANVLTNELRLIAQRYLL	61	bMAPKAPK3-146	IMRDIGTAIQFLHSHNIA
30	bTCN2-2	RHLGAFLFLLGVLGALTE	62	bMAPKAPK3-275	EDAKQLIRLLLKTDPTER
31	bTCN2-47	LEHLNPSIYVGLRLSSLQ	63	bMAPKAPK3-303	WINQSMVVPQTPLHTARV
32	bTCN2-217	YSTPLALQFLMTSPMRGA			

A total of 63 peptides were predicted to be epitopes derived from the 34 antigen proteins. The numbers in the peptide names represent the first amino acid number of the original protein. b: biotin-tag.

In the second screening, these 63 peptides were synthesized and used as antigens for the analysis of the serum antibody levels by AlphaLISA using sera from 48 CTEPH patients and 48 HDs. The levels of autoantibodies for nine peptides (No. 8, 10, 11, 19, 34, 35, 43, 46, 52) seemed higher in CTEPH patients than in HDs. We then performed an additional AlphaLISA analysis for those 9 autoantibodies using sera from 96 CTEPH patients and 96 HDs. These patients’ basic characteristics are summarized in Table 3.

**Table 3:** Basic characteristics of patients whose sera were used in the AlphaLISA analysis.

	CTEPH (n = 96)	PAH (n = 65)	Healthy Donor Control (n = 96)
Sex (M:F)	19:77	18:47	53:43
Age	59.9±11.2	51.0±17.6	56.2±9.28
Height (cm)	157.6±10.0	159.1±8.9	164.1±8.55
Body weight (kg)	56.3±12.0	57.3±14.9	63.4±11.8
Body mass index	22.5±3.2	22.4±4.43	23.4±3.48
Systolic blood pressure (mmHg)	125.1±20.5	124.9±23.8	120.4±16.0
Diastolic blood pressure (mmHg)	75.3±13.8	70.6±11.5	78.1±11.4
Mean pulmonary artery pressure (mmHg)	44.4±11.4	41.8±10.4	
Pulmonary Vascular Resistance (dyne·sec·cm^-5^)	700.4±311.3	606.0±279.7	
Cardiac output (L/min)	4.22±1.17	4.67±1.27	
Cardiac index (L/min/m^2^)	2.95±0.77	2.86±0.78	
WHO-FC (I:II:III:IV)	0:47:47:2	0:47:18:0	

**Fig 1:**
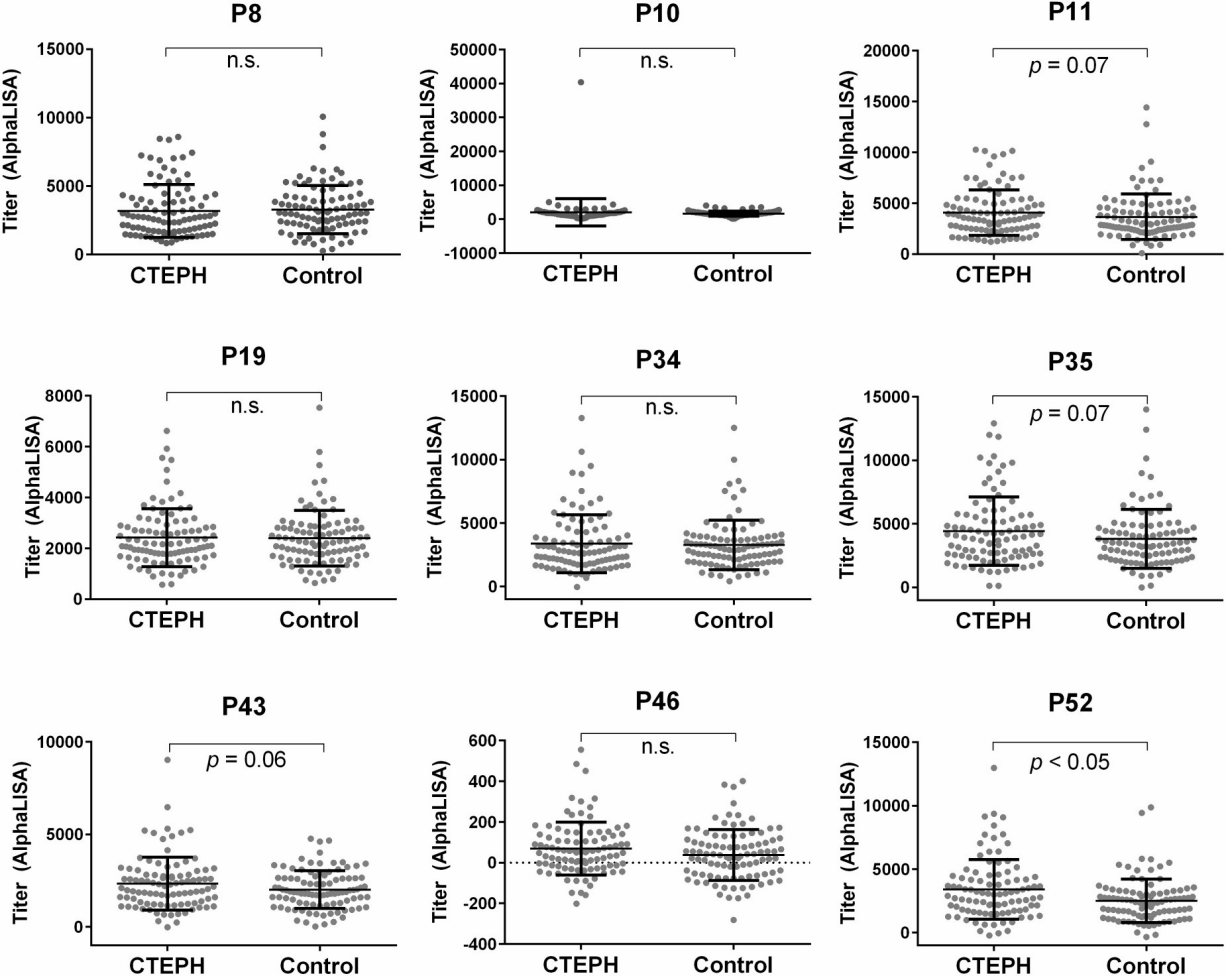
A comparison of the serum antibody levels against nine crude synthetic peptides between CTEPH patients and HDs. The results of the AlphaLISA analyses are shown. The Y-axis shows AlphaLISA counts which represent antibody levels (titer) in sera. The error bars show the mean and standard deviation. n.s.: not significant.

Finally, the serum levels of autoantibodies against four peptides (No. 11, 35, 43, 52) seemed higher in CTEPH patients than in HDs (Fig 1) (A statistically significant difference in the auto-antibody level was noted for peptide No. 46 between the two groups; however, both groups had an extremely low alpha count, so the difference was not deemed to be etiologically significant).

The selected antigenic peptides were as follows:

No. 11: bC9orf163-64, biotinylated peptide of amino acid positions 64–84 of C9orf163
No. 35: bAPOBEC4-312, biotinylated peptide of amino acid positions 312–329 of APOBEC4
No. 43: bEXD2-307, biotinylated peptide of amino acid positions 307–324 of EXD2
No. 52: bPHAX-247, biotinylated peptide of amino acid positions 247–264 of PHAX

### Third screening using purified peptide

**Fig 2:**
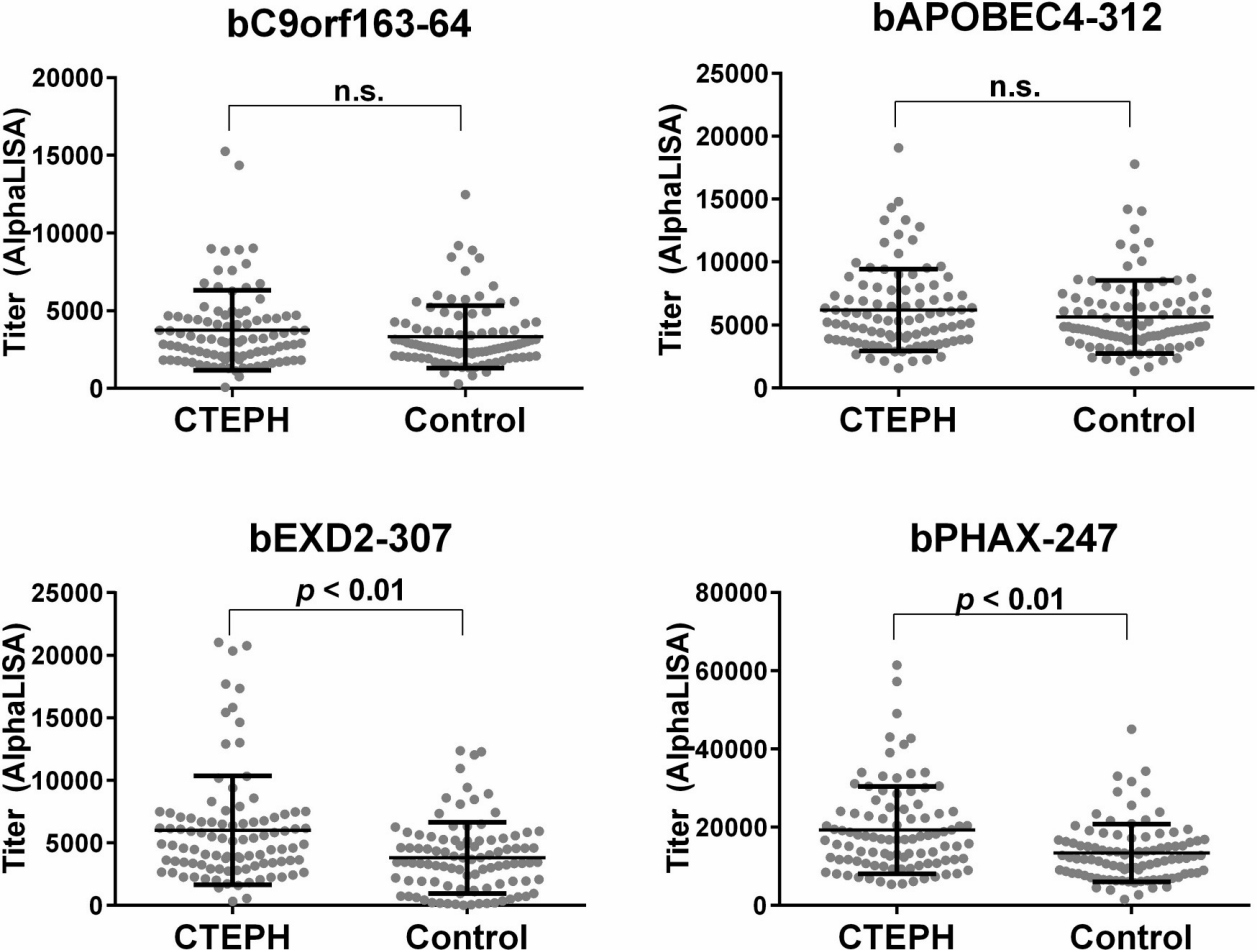
A comparison of the serum antibody levels against four purified synthetic peptides between CTEPH patients and HDs. The results of the AlphaLISA analyses are shown. The error bars show the mean and standard deviation. n.s.: not significant.

We then obtained purified antigenic peptides of bC9orf163-64, bAPOBEC4-312, bEXD2-307, and bPHAX-247, of which the purity was 98.10%, 95.59%, 95.43%, and 91.19%, respectively. We performed an AlphaLISA analysis using those 4 purified peptides with the same sera of 96 CTEPH patients and 96 HDs as a third screening. We detected significantly higher serum levels of antibodies against bEXD2-307 and bPHAX-247 in CTEPH patients than in HDs (Fig 2).

### Correlation between antibodies’ titers and clinical parameters in CTEPH patients

**Fig 3:**
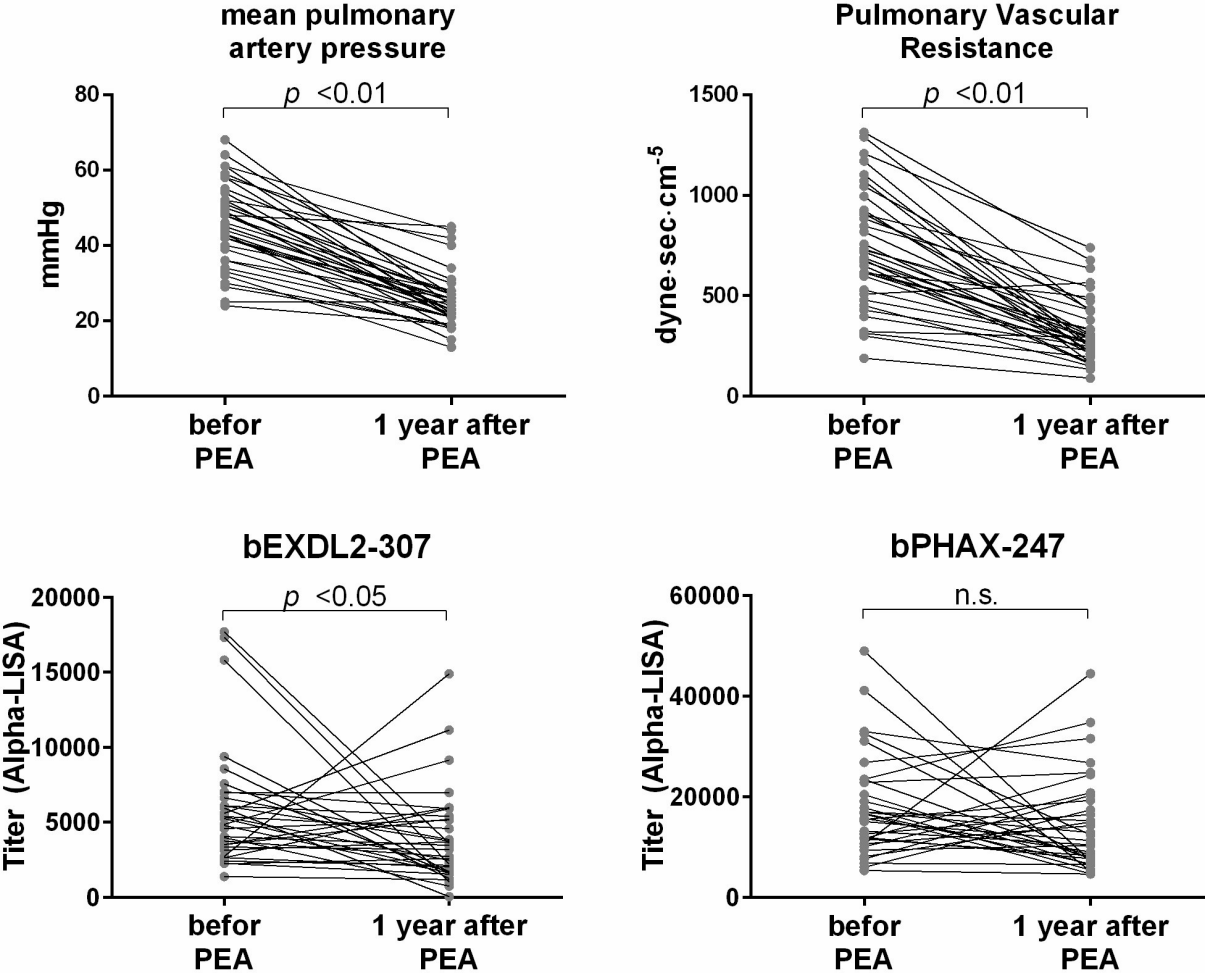
The pulmonary hemodynamics and the fluctuation in the serum antibody levels against EXD2 and PHAX peptides in patients with CTEPH before and one year after pulmonary endarterectomy (PEA). The mean pulmonary artery pressure decreased from 45.5±10.9 to 25.3±7.4 mmHg and the pulmonary vascular resistance decreaseased from 732.0±287.4 to 322.1±157.2 dyne·sec·cm^-5^. The titers of EXD2 also decreased after these successful PEA. n.s.: not significant.

We then analyzed correlations between the antibody levels and the clinical data of the patients, including the gender, age, body mass index, symptom duration, and hemodynamic parameters measured by right heart catheter. We found the antibody levels of bEXD2-307 showed a mild negative correlation between the arterial oxygen pressure in CTEPH patients (Table 4) and did decrease after successful pulmonary endarterectomy in CTEPH patients (Fig 3).

**Table 4:** Correlation coefficient between autoantibody titers and clinical parameters in CTEPH patients.

Factors	Correlation Coefficient	*p* value
bEXD2-307		
Age	0.1094	0.2886
Symptom duration (months)	-0.0100	0.9285
Mean pulmonary artery pressure (mmHg)	0.1227	0.2338
Pulmonary vascular resistance (dyne·sec·cm^-5^)	0.0656	0.5279
Cardiac output (L/min)	0.0220	0.8313
Arterial oxygen pressure (mmHg)	-0.2096	0.0415 †
Mixed venous oxygen pressure	-0.1074	0.3004
bPHAX-247		
Age	0.1075	0.2972
Symptom duration (months)	-0.0419	0.7070
Mean pulmonary artery pressure (mmHg)	0.0154	0.8818
Pulmonary vascular resistance (dyne·sec·cm^-5^)	0.0140	0.8929
Cardiac output (L/min)	0.0165	0.8732
Arterial oxygen pressure (mmHg)	-0.0177	0.8647
Mixed venous oxygen pressure	-0.0130	0.9003

†: *p* < 0.05. EXD2: exonuclease 3'–5' domain-containing 2; PHAX: Phosphorylated Adaptor for RNA Export.

### An exploratory analysis of the autoantibodies in PAH and SAS patients

**Fig 4:**
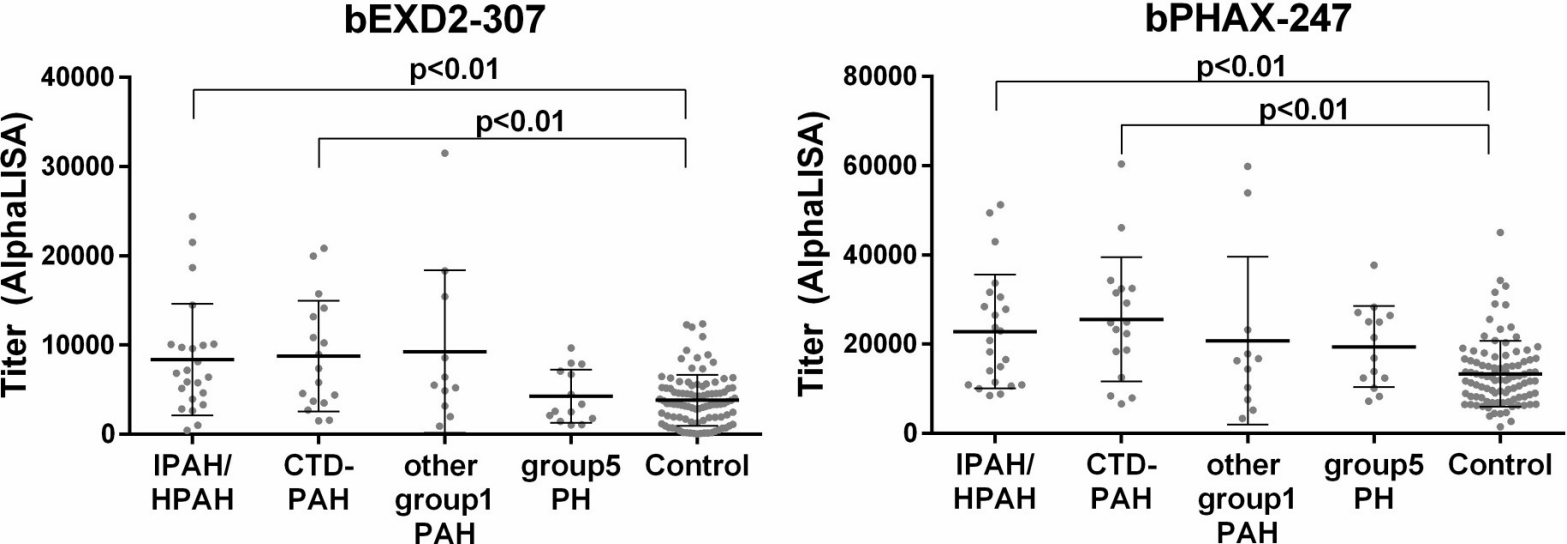
A comparison of the serum antibody levels against EXD2 and PHAX peptides among subgroups of PAH and HDs. CTD-PAH: PAH with connective tissue disease. Group 1 PAH: congenital heart disease, portopulmonary hypertension. Group 5 PH: pulmonary artery stenosis, aortitis, sarcoidosis. The error bars show the mean and standard deviation.

As an exploratory analysis, we examined the serum antibody levels for those two purified peptides using another set of sera from patients with PAH. The serum antibody levels to bEXD2-307 and bPHAX-247 were also elevated in patients with PAH, similar to our observations in CTEPH patients. A sub-analysis of sub-groups of PAH revealed that the patients with idiopathic PAH (IPAH) and PAH with connective tissue disease (CTD-PAH) had significantly higher titers for those peptides than HDs (Fig 4). The antibody levels of bEXD2-307 and bPHAX-247 did not correlate with pulmonary hemodynamics (data not shown). We were able to measure the level of auto-antibody after the treatment with selective pulmonary vasodilator in 17 of the 65 PAH patients (treatment naïve: n = 4, add-on: n = 13; mean treatment period: 33.1±13.5 months). The mean pulmonary artery pressure decreased from 45.3±9.6 to 40.1±13.8 mmHg, but the pulmonary vascular resistance did not decrease. The titers of anti-EXD2 antibody and anti-PHAX antibody did not decrease after the treatment neither (S1 Fig).

We also examined the titers of those 2 auto-antibodies in 80 patients with SAS, another hypoxic condition. Their mean age was 58.7±11.0 years, and the sex ratio (M:F) was 54:26. The apnea/hypopnea index (AHI) in these patients was as follows: mild (5–15) in n = 11, moderate (15–30) in n = 17, and severe (30 or higher) in n = 52. However, there was no significant difference in titers between HDs and SAS patients (S2 Fig).

## Discussion

We screened for autoantibodies in the sera of CTEPH patients using ProtoArray, which resulted in the selection of 34 candidate antigens (Table 1). We then synthetized 63 crude peptides predicted to be the epitope sites of those 34 candidate antigens (Table 2) and evaluated the titers of the autoantibodies for those peptides by AlphaLISA (Fig 1). Based on these results, we synthetized the four purified peptides and re-evaluated the titer of the autoantibodies against those four peptides by AlphaLISA. We ultimately found that CTEPH patients had high titers of autoantibodies for exonuclease 3'-5' domain-containing 2 (EXD2) and phosphorylated adaptor for RNA export (PHAX) than HDs (Fig 2). Furthermore, the antibody levels for EXD2 showed a mild negative correlation between the arterial oxygen pressure in CTEPH patients and decreased after successful pulmonary endarterectomy in CTEPH patients (Fig 3). As an exploratory analysis, we examined the serum antibody levels for those four purified peptides using another set of sera from patients with PAH and SAS. The serum antibody levels to EXD2 and PHAX were also elevated in patients with PAH, similar to our findings in CTEPH patients (Fig 4). Thus, EXD2 and PHAX may be markers associated with PH in general and not specific to CTEPH.

EXD2 is a 621-amino acid protein with a nuclease domain [15]. Its precise role had been unclear; however, Ronan et al. reported that EXD2 is an exonuclease essential for DNA double-strand break (DSB) resection and efficient homologous recombination. EXD2 functionally interacts with the Mre11-Rad50-Nbs1 (MRN) complex to accelerate resection via its 3'-5' exonuclease activity, which efficiently processes double-strand DNA substrates containing nicks [16]. EXD2 also reportedly has a critical functional link with FANCD2, the gene responsible for Fanconi anemia [15]. Fanconi anemia is a severe, congenital form of anemia associated with disorder of DNA repair. It should be noted that the serum anti-EXD2 antibody levels showed mild inverse correlation with arterial oxygen pressure (*p* = 0.0415) (Table 4). DSBs can be induced by ischemia such as anemia and low oxygen pressure [17]. Thus, it is possible that EXD2 required for repair of DSBs is expressed to the elevated levels, which may induced the development of its autoantibodies.

PHAX is a key molecule in transporting small nuclear RNA (snRNA) and small nucleolar RNA (snoRNA) from the nucleus to the cytosol. PHAX configures a complex with RNA, CBP80, and CBP20 known as the cap binding complex (CBC), followed by binding with CRM1/RanGTP, which leads to the export of RNA into the cytoplasm [18, 19]. snRNAs and snoRNAs play an essential role in the maturation of mRNAs and rRNAs, respectively [20].

As described above, EXD2 and PHAX are reported to be involved in DNA repair and the transportation of snRNA/snoRNA, respectively. The precise mechanisms underlying the production of autoantibodies for these antigens were not determined in the present study; however, the titers of those antigens were elevated in both CTEPH and PAH patients, suggesting that this autoantibody production may share a common etiology in CTEPH and PAH patients, e.g. distal pulmonary artery remodeling or right heart burden. It is reported that oxidative stress and inflammation contribute to vascular remodeling [21], and these factors are also known to favor DNA damage [22]. Indeed, high levels of DNA damage were reported to occur in PAH lungs and remodeled arteries [23] [24] [25]. These previous findings suggest that PH-induced DNA damage followed by the activation of the DNA repair system might account for the overexpression of these antigens and the resultant development of autoantibodies. Regarding DNA damage, both the polyadenylation and deadenylation of mRNA are markedly affected via CBP80 and CBP20 [26]. Thus, the functional roles of EXD2 and PHAX may be interrelated. Further investigations focused on the production of these autoantibodies might give us information on the etiology of CTEPH and PAH as well as prove the utility of these autoantibodies as a new diagnostic tool.

Our study had several limitations. First, as the controls were healthy volunteer donors, potential confounding factors between patients with CTEPH and controls (e.g. the age, BMI, hypertension, diabetes, and hyperlipidemia) were not adjusted in the analysis in the current study. Second, we did not investigate the exact mechanism underlying the autoantibody production, so we were unable to determine the pathological etiology of this phenomenon. Third, this study was performed retrospectively at a single institution in Japan. Additional prospective, multicenter investigations are therefore required.

In conclusion, patients with CTEPH and PAH had higher titers for autoantibodies to EXD2 and PHAX than HDs. These autoantibodies may provide novel useful diagnostic and therapeutic tools for treating those patients.

## Supporting information

S1 Fig The pulmonary hemodynamics and titers of anti-EXD2/anti-PHAX antibody before and after the treatment with selective pulmonary vasodilator in PAH patients. The mean pulmonary artery pressure decreased from 45.3±9.6 to 40.1±13.8 mmHg, but the pulmonary vascular resistance did not decrease. The titers of anti-EXD2 antibody and anti-PHAX antibody did not decrease after the treatment neither.
(TIF)

S2 Fig The titers of anti-EXD2 antibody and anti-PHAX antibody in patients with sleep apnea syndrome. There was no significant difference in the titers of anti-EXD2 antibody or anti-PHAX antibody between the patients with sleep apnea syndrome and HDs.
(TIF)
